# The Neurobase of ambiguity loss aversion about decision making

**DOI:** 10.3389/fpsyg.2023.1055640

**Published:** 2023-01-26

**Authors:** Yiqin Hu, Xinbo Lu, Wanjun Zheng, Luting Wang, Ping Yu

**Affiliations:** ^1^School of Economics, Zhejiang University of Finance and Economics, Hangzhou, China; ^2^Center for Economic Behavior and Decision-Making (CEBD), Zhejiang University of Finance and Economics, Hangzhou, China; ^3^School of Economics, Jiaxing University, Jiaxing, China

**Keywords:** ambiguity loss aversion, ambiguity gain, dorsolateral prefrontal cortex, transcranial direct current stimulation, decision-making

## Abstract

In our daily decision-making, there are two confusing problems: risk and ambiguity. Many psychological studies and neuroscience studies have shown that the prefrontal cortex (PFC) is an important neural mechanism for modulating the human brain in risk and ambiguity decision-making, especially the dorsolateral prefrontal cortex (DLPFC). We used transcranial direct current stimulation (tDCS) to reveal the causal relationship between the DLPFC and ambiguity decision-making. We design two experimental tasks involving ambiguity to gain and ambiguity to loss. The results of our study show that there is a significant effect on left DLPFC stimulation about ambiguity to loss, there is an insignificant effect on left DLPFC stimulation about ambiguity to gain, and there is an insignificant effect on right DLPFC stimulation about ambiguity to gain and ambiguity to loss. This result indicates that people are more sensitive to ambiguity loss than ambiguity gain. Further analysis found that the degree of participants’ attitudes toward ambiguity loss who received anodal simulation was lower than that who received sham stimulation across the left DLPFC, which means that the subjects had a strong ambiguity loss aversion after the participants received the anodal simulation of the left DLPFC.

## 1. Introduction

In our daily decision-making, there are two puzzling problems: risk and ambiguity. Just as their names imply, risk is a combination of the possibility and consequences of a specific dangerous situation, that is, the possibility of consequences that we do not want. Ambiguity is the uncertainty of “whether it is nor non” or “like” or “specious” in the judgment of things, which is the uncertainty of confirmation of things. In summary, risk is the world we know; that is, the probabilities of outcomes are determined, such as the gamble of coin tossing. While ambiguity is the world we do not know; that is, the probability of events are uncertain, such as whether it will rain tomorrow.

The research on the psychology of risk and ambiguity mainly focuses on risk preferences and ambiguity aversion, further extending loss aversion. Knight (1921) classified uncertainty decisions: one of the extremes of certainty is “ignorance,” which occurs when the possibility of the result is completely unknown; the other is “risk,” which occurs when the probability of the result is already known. “Ambiguity” is between the above two, and occurs when the probability of the result is incompletely known. Ellsberg (1961) found that people have ambiguity aversion tendencies in bottle selection tasks. Subsequently, an increasing number of scholars (Camerer and Weber, 1992; Fox and Weber, 2002; Dijk and Zeelenberg, 2003) have found that people generally tend to be ambiguity averse. Kahneman and Tversky (1979), Tversky and Kahneman (1991) mentioned the influencing factor of loss aversion when they proposed prospect theory. Loss aversion is an important point in prospect theory, which shows that investors value the importance of “avoiding harm” over “profit-making.”

The neural mechanism of social cognition has become a hot topic in cognitive neuroscience research. The risk decision-making ability is an important part of social cognitive functions. Research on brain functional imaging suggests that amygdala, prefrontal cortex, and ventral striatum may participate in the cognitive plus of decision-making work process (Adolphs et al., 2002; Ernst et al., 2005; Asaad and Eskandar, 2011; Deserno et al., 2015). The frontal cortex is one of the most developed brain areas of human beings, not only participating in memory, attention, emotion, and other cognitive activities in society but also playing an important role in risk decision-making. Uncertainty of gain and loss may lead to some negative consequences for decision makers or may be a new opportunity for decision makers. Exploring and understanding how people make effective decisions and the cognitive neurological mechanisms behind various risks have become hotspots in psychology, economics, finance, and management in recent years.

Currently, neuroscientific researchers have found that the dorsolateral prefrontal cortex (DLPFC) and the orbital frontal cortex (OFC) influence human uncertainty decision-making (Krain et al., 2006; Yang et al., 2017). The study conducted by Krain et al. (2006) suggested that the activity of DLPFC impacts the decision-making regarding ambiguity, while the activity of OFC impacts the decision-making about risk, whose method by using the meta-analysis. In contrast, a study performed by Yang et al. (2017) showed that the DLPFC influences risk decision-making, whereas the OFC impacts ambiguity decision-making through transcranial direct current stimulation (tDCS). In addition, some studies have found that there is a positive relationship between the right DLPFC and risky decision-making (Ye et al., 2015a,b, 2016; Huang et al., 2017), while other studies found negative or insignificant results (Knoch et al., 2006; Fecteau et al., 2007a,b).

These inconsistent results may have originated from the different experimental tasks used, such as Rogers’ Risk Task (Rogers et al., 1999), the Balloon Analog Risk Task (BART), and the risk measurement table (Ye et al., 2015b). Furthermore, the shortage of research by Yang et al. (2017) is that its brain stimulation adopts bilateral stimuli, and the positioning of these brain areas is not sufficiently accurate. Therefore, our experimental task was based on Cardenas and Carpenter (2013) because our experimental purpose is to study the brain area of ambiguity aversion in the frame of gain and loss. Then, we simplified the experimental tasks and distinguish the two types of ambiguity gain and ambiguity loss, and we choose unilateral stimuli on the DLPFC. As a result, we can make a clearer judgment of DLPFC activity in response to ambiguity aversion. In addition, Huang et al. (2017) conducted unilateral stimulation of the DLPFC for risky decision-making based on gain and loss frames, and then, we also chose the stimulus brain area of the DLPFC to research the relationship between the targeted brain area and ambiguity regarding gains and losses. Although Abdellaoui et al. (2016) studied how to measure the event weights in ambiguity decisions, they subsequently researched how to measure the event loss under risk decisions, and Abdellaoui et al. (2016) showed the loss aversion in ambiguity decisions. Although some neuropsychology studies or neuroscience studies using the Iowa Gambling Task (IGT) to research ambiguity decisions under the frames of gains and losses, their results were different. Bechara et al. (1994) found that the ambiguity decisions influenced by prefrontal cortex (PFC). Subsequently, Bechara et al. (2000) showed that the ventromedial prefrontal cortex (vmPFC) had impacted on the ambiguity decisions. While Fellows and Farah (2005) suggested that not only vmPFC, but also DLPFC were related to the performance on the IGT about ambiguity decisions. We found that the ambiguity decisions impacted by different brain regions under different tasks. Even for the same task, the brain regions influenced ambiguity decisions may also be different. However, there was no study on how neural mechanisms on ambiguity decisions were made in the frame of gains and losses through tDCS technology, which our paper achieves.

## 2. Materials and methods

### 2.1. Subjects

We recruited 111 subjects taking part in our experiment. They were right-handed healthy students who had no history of clinical impairments, neurological disorders, or psychiatric problems. All participants came from Zhejiang University of Finance and Economics; most of them were undergraduates, and a few were postgraduates. Their average age was 21 (SE = 1.66), and their ages ranged from 18 to 26. Our experimental subjects included 52 males and 51 females who were naïve to tDCS and our ambiguity choice tasks. The participants’ payments contained a fixed show-up fee of 10 RMB (~ 1.48 US dollars) plus the gain and loss from the ambiguity choice tasks. On average, subjects received approximately 45 RMB (~ 6.67 US dollars) after the experiment finished according to their performance and the computer program. The entire experiment lasted 1 h. Our experiment was approved by the Zhejiang University of Finance and Economics Ethics Committee. Before the experiment started, the participants were asked to provide written informed consent. None of the participants reported any adverse side effects of the scalp or headache pain during or after this experiment.

### 2.2. tDCS

As a popular neuromodulatory technique, tDCS has been applied to excite or inhibit the cerebral cortex by a mild direct electrical current (e.g., 1–2 mA; Brunoni et al., 2012) and in human noninvasive brain stimulation for over 20 years (Priori et al., 1998); it can modulate spontaneous neuronal activity, as shown by Fritsch et al. (2010). It was used to increase the cortical excitability of the targeted brain region and caused no physiological harm to the subjects. It was attached to the scalp through two saline-soaked surface sponge electrodes (one was 3 cm*3 cm, and the other was 5 cm*7 cm) to change the critical level of excitability. Generally, when anodal stimulation is applied, cortical excitability is increased, while cathodal stimulation is performed, and cortical excitability is decreased (Nitsche and Paulus, 2000). The technology of tDCS is always applied in psychological research for specific brain areas or specific psychological problems. In this paper, we studied the impact of a tDCS device (NeuroConn, Ilmenau, Germany) on the cortical excitability of the DLPFC.

For the left DLPFC tDCS treatments, the participants were randomly allocated one of the two stimulation types: 20 males and 19 females (*n* = 39) received anodal stimulation, their average age is 21.08(1.84), and 16 males and 20 females (*n* = 36) received sham stimulation, their average age is 20.94(1.75). For the right DLPFC tDCS treatments, the participants were randomly allocated one of the two stimulation types: 16 males and 20 females (*n* = 36) received anodal stimulation, their average age is 20.74(1.39), and 16 males and 20 females (*n* = 36) received sham stimulation, their average age is 20.94(1.75). Furtherly, the Kruskal-Wallis test showed that there were no differences between age across the treatments (*χ^2^_d.f.2_* = 0.616, *p* = 0.735). And the decision tasks of ambiguity loss and gain were between-subject design.

**Figure 1:**
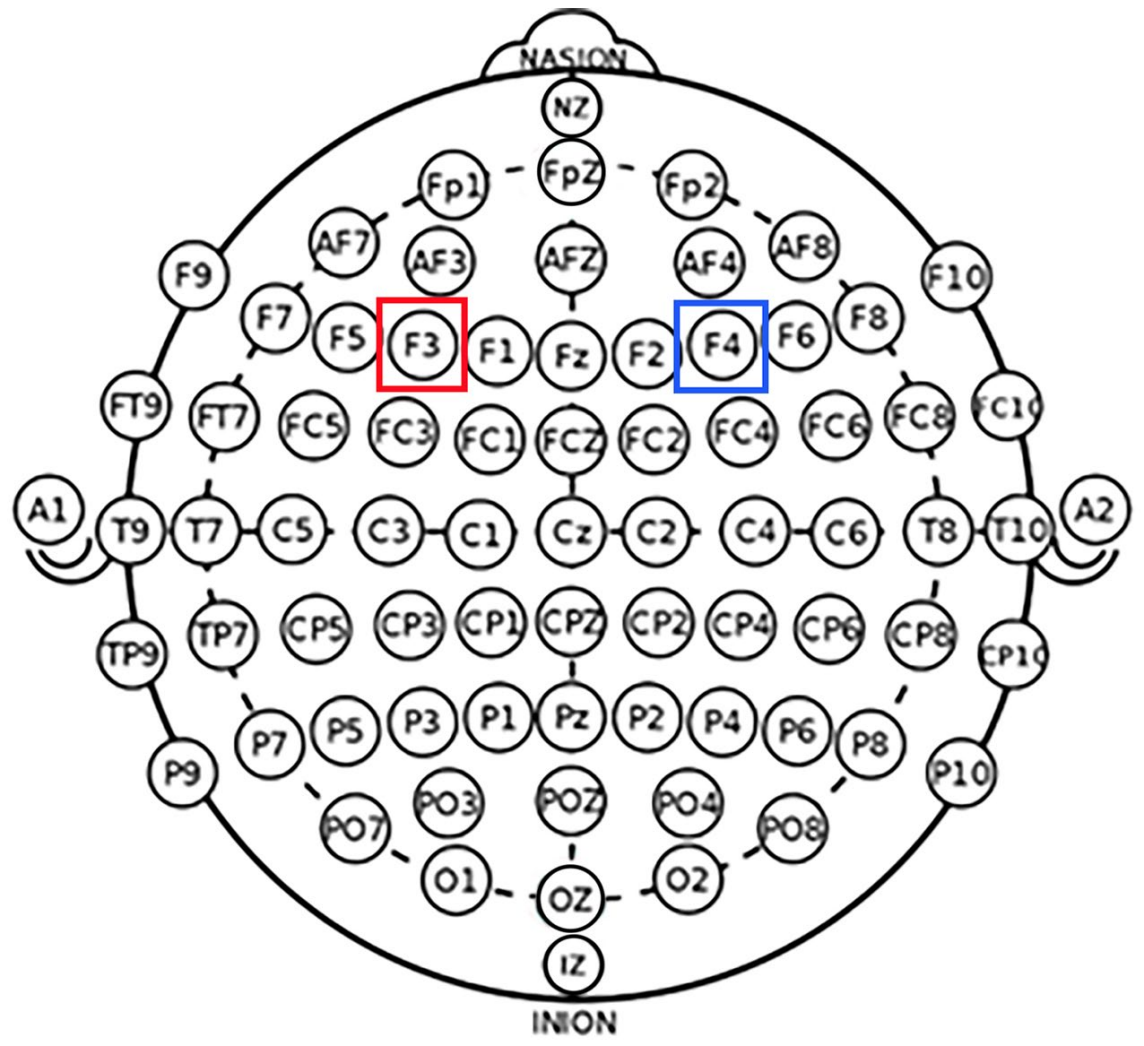
Schematic and electrode locations of DLPFC. Schematic of the electrode positions F3 and F4 based on the international EEG 10–20 system.

**Figure 2:**
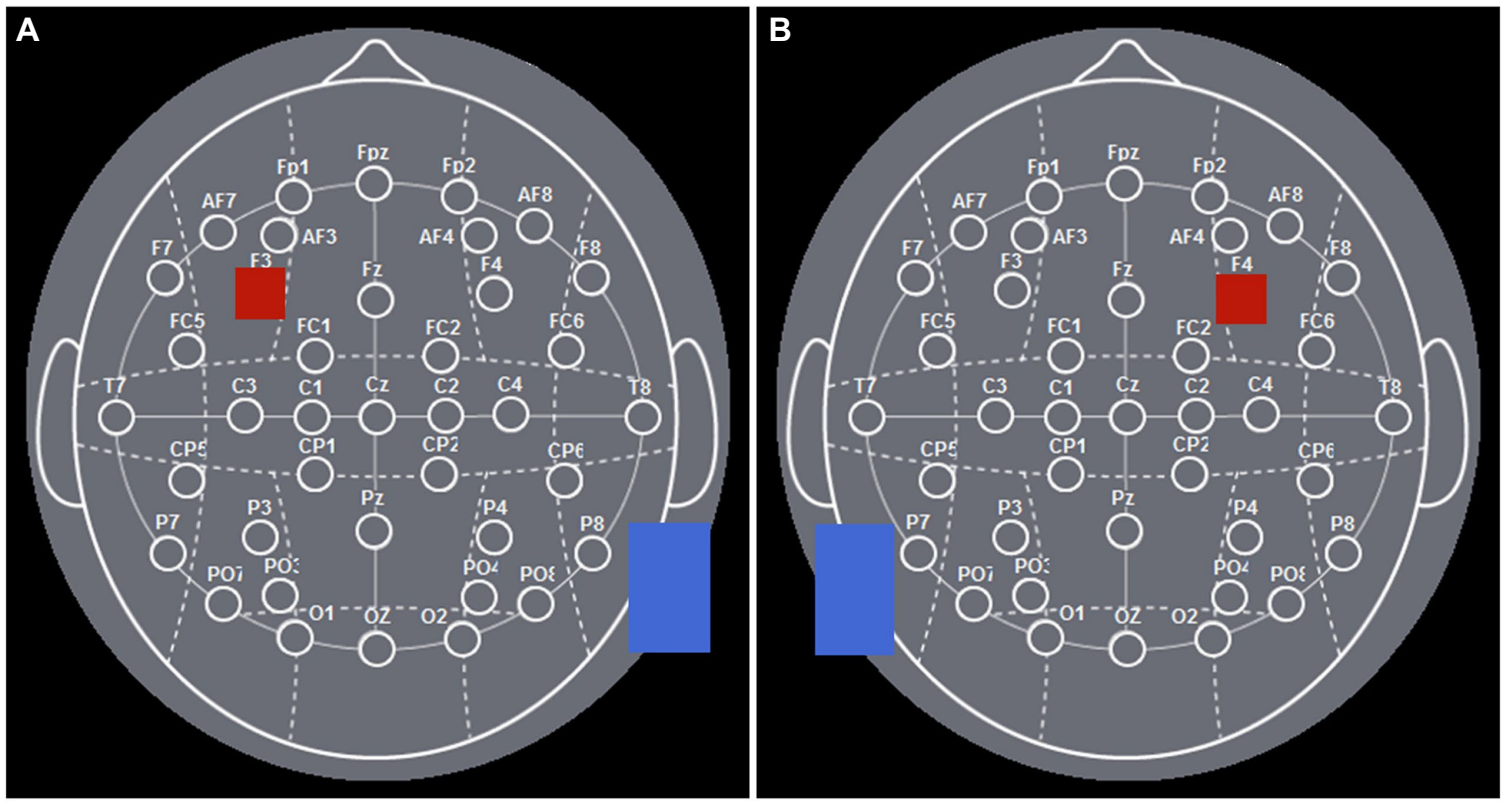
The electrode positions of DLPFC. **(A)** The electrode positions of left DLPFC. **(B)** The electrode positions of right DLPFC.

According to the international EEG 10–20 system, the left DLPFC was positioned over F3, while the right DLPFC was positioned over F4 (Figure 1). Learned from Yang et al. (2021), the experiment was performed as follows: for the left DLPFC tDCS stimulation, we placed the anodal electrode (3 cm*3 cm) over F3 to achieve the anodal stimulation while placing the cathodal electrode (5 cm*7 cm) over the cheek (Figure 2A). For the right DLPFC tDCS stimulation, we placed the anodal electrode (3 cm*3 cm) over F4 to achieve the anodal stimulation while placing the cathodal electrode (5 cm*7 cm) over the cheek (Figure 2B).

The tDCS lasted 20 min, stimulating with a current of 1.5 mA to the targeted brain area, fading 30 s in and fading 30 s out (Dymond et al., 1975). Nitsche and Paulus (2000) and Nitsche et al. (2003) previously improved the protection and reliability of tDCS in their experiments. For the sham stimulation, the procedure was similar to the anodal stimulation, but the current was different in lasting only 30 s. Even though the participants initially felt itchy, during the remaining stimulation time, no current was passed, so they thought that they had received the same stimulation as others. This approach has been proven reliable because the limited stimulation of short-term duration on the targeted area hardly modulates cortical excitability (Gandiga et al., 2006). To ensure that the subjects had no idea which type of stimulus they had received, we implemented the processes identically after setting the device parameters before the experiment. Besides, because of the sham stimulation does not play the role of modulating the activity of left DLPFC or right DLPFC, so the locations of the electrodes of sham stimulation were placed on the left DLPFC and the cheek as the combined sham treatments.

## 3. Experimental design

Our experiment was designed by Cardenas and Carpenter (2013) and adapted to our research purpose. We made some changes to their experimental task about ambiguity: (1) we set two tasks to assess the participants’ attitudes toward ambiguity loss and ambiguity gain separately. (2) Similar to their lottery games, we also set up six games and asked the participants to choose one of six games to take part in. Each game contains two probabilities: 30 and 70%. (3) For the ambiguity loss task, we first give 95 experimental currency units to participants to reduce their worries about their experimental payments; then, we ask them to make unbiased decisions. (4) Different from the lottery games designed by Cardenas and Carpenter (2013), we used 6 text expressions for the participants to choose from to measure the degree of subjects’ ambiguity aversion more simply and clearly. It should be noted that each participant had to complete both tasks in the gain and loss frames, and the order of the two tasks was random. After the subjects finished the whole experiment tasks, the computer would show the subjects the experimental reward, respectively.

### 3.1. The decision task used to assess participants’ attitudes toward ambiguity loss

In this experimental task, the participants are asked to take part in one of the six games. Each game contains two possible results. The task is as follows:

At the beginning of this game, you have 95 experimental currency units (ECUs). Of the following possibilities, one is 30% likely, and the other is 70% likely. Please make your choice:

1. You lose 62 ECUs.
2. You may lose 70 ECUs or may lose 48 ECUs.
3. You may lose 77 ECUs or may lose 33 ECUs.
4. You may lose 84 ECUs or may lose 18 ECUs.
5. You may lose 91 ECUs or may lose 4 ECUs.
6. You may lose 95 ECUs or may lose 0 ECUs.

If you choose the first option, you lose 62 ECUs. If you choose the second game, you may lose 70 ECUs at a probability of 30% or 70%, or you may lose 48 ECUs at a probability of 30% or 70%. The third game to sixth game are the same.

### 3.2. The decision task used to assess attitudes toward ambiguity gain

In this experimental task, the participants are asked to take part in one of the six games. Each game contains two possible results. The task is as follows:

In this game, the following possibilities are 30% or 70% likely. Please make your choice:

1. You gain 33 ECUs.
2. You may gain 25 ECUs or may gain 47 ECUs.
3. You may gain 18 ECUs or may gain 62 ECUs.
4. You may gain 11 ECUs or may gain 77 ECUs.
5. You may gain 4 ECUs or may gain 91 ECUs.
6. You may gain 0 ECU or may gain 95 ECUs.

If you choose the first game, you gain 33 ECUs. If you choose the second game, you may gain 25 EUC at a probability of 30% or 70%, or you may gain 47 ECU at a probability of 30% or 70%. The third game to sixth game are the same.

### 3.3. Procedure

**Figure 3:**
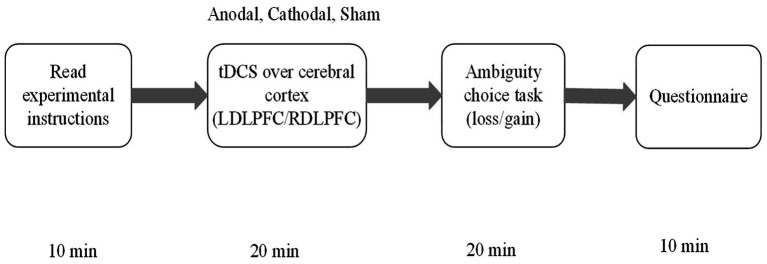
The experimental procedure.

Experimental software z-tree is used to present the two tasks about ambiguity loss and ambiguity gain as well as to automatically calculate our experimental data and the participants’ final payoff (Fischbacher, 2007). Before the task, the participants are randomly arranged in a seat and stimulated by a tDCS instrument for 20 min. Our formal experiment will last 20 min. After the formal experiment is finished, the participants are asked to complete a questionnaire, which took 10 min. The questionnaire asked about the participants’ personal information, such as age, gender, city, father’s education, mother’s education, and income. After the questionnaire was completed, the participants could receive their payments from our experimenter. The payments contained the participants’ ambiguity task earnings and their fixed show-up fees. Figure 3 illustrates the procedure of the entire experiment.

## 4. Data analysis

First, we performed regression analysis to assess the tDCS stimulation influences of the left DLPFC and right DLPFC on subjects’ attitudes toward ambiguity loss and ambiguity gain. The stimulation of the left DLPFC included anodal left DLPFC/cathodal cheek and sham. The stimulation of the right DLPFC included anodal right DLPFC/cathodal cheek and sham. The equation of each participant i is as follows:


$$y=\beta_{0}+\beta_{1}^{*}D_{i}+\beta_{2}^{*}X_{j}+\varepsilon_{i}$$

where y is the dependent variable, which denotes the participant’s attitudes toward ambiguity loss/ambiguity risk. The participants’ attitudes toward ambiguity gain/loss coded from 1 to 6, where 1 represents choosing the first game, and 6 represents choosing the last game in our experimental task. $D_{i}$ are dummy-coded variables that are set to 1 if participant i received anodal stimulation, including left DLPFC and right DLPFC, and the parameters $\beta_{1}$ quantify the change in the degree of the participant’s attitudes toward ambiguity loss/ambiguity gain due to tDCS stimulation relative to the sham group. Furthermore, we add the participants’ personal information, such as age, gender, and income as the control variables to capture the effects of personal characteristics. In our regressions, the gender as dummy-coded variable that are set to 1 if participants are male. While age and income take the actual values reported in the questionnaire.

Second, after the impact of stimulation was robust *via* regression analysis, then we used two-sample Wilcoxon rank-sum (Mann–Whitney) tests to assess the differences between the degrees of the participants’ attitudes toward ambiguity loss/ambiguity gain obtained from different types of stimulation. The purpose of our experiment was to examine whether tDCS stimulation of left/right DLPFC activity impacted the participants’ attitudes toward ambiguity loss/ambiguity gain. Therefore, we hypothesized that there would be a significant effect in the stimulation comparison.

Finally, SPSS and STATA software are used to statistically evaluate all our experimental data. We set *p* < 0.05 for the critical level of significance for all analyses. The means (M) and standard errors (SE) of the data for the degree of the participants’ attitudes toward ambiguity loss/ambiguity gain under different stimulation conditions of the left/right DLPFC are shown in Table 1. It should be noted that the degree of the participants’ attitudes toward ambiguity loss/ambiguity gain is based on their choices. In our experiment, if they choose the first game, it means that they are ambiguity averse; as the game serial number increases, the subjects prefer ambiguity. If they choose the last game, it means that the subjects have an ambiguity preference.

**Table 1:** Mean (M) and standard error (SE) of the dataset about the degree of attitudes toward ambiguity loss/gain under stimulation conditions.

	Ambiguity loss	Ambiguity gain
anodal_LDLPFC	3.25*** (0.25)	2.89 (0.28)
anodal_RDLPFC	3.77 (0.25)	2.97 (0.28)
Sham	4.11 (0.24)	3.08 (0.3)

Standard error in brackets; ***p < 0.001, **p < 0.01, *p < 0.05, the *p* value reflects the significance of the results of Wilcoxon rank-sum tests among treatments compared to sham treatment.

## 5. Results

### 5.1. Descriptive statistics

In Table 1, first, we found that the mean degree of the attitudes toward ambiguity loss among those who received anodal stimulation was lower than that among those who received sham stimulation, regardless of the left DLPFC tDCS or the right DLPFC tDCS, while the standard error of attitudes toward ambiguity loss among those who received anodal stimulation was higher than that among those who received sham stimulation, regardless of the left DLPFC tDCS or the right DLPFC tDCS. Second, we can find that the mean degree of the attitudes toward ambiguity gain among those who received anodal stimulation is lower than that among those who received sham stimulation, no matter to the left DLPFC tDCS or the right DLPFC tDCS, while the standard error of attitudes toward ambiguity risk among those who received anodal stimulation is higher than that among those who received sham stimulation, no matter to the left DLPFC tDCS or the right DLPFC tDCS. Third, we find that the mean degree of attitudes toward ambiguity loss is higher than ambiguity gain under three stimulation types: anodal left DLPFC stimulation, anodal right DLPFC stimulation, and sham stimulation. The standard error of attitudes toward ambiguity loss is lower than that of attitudes toward ambiguity risk under three stimulation types. Finally, we can conclude that our participants had stronger attitudes toward ambiguity loss aversion than toward ambiguity gain aversion. Furthermore, we also suggest that compared to those receiving sham stimulation, the participants’ attitudes toward ambiguity loss or ambiguity gain are decreased under anodal left DLPFC stimulation and anodal right DLPFC stimulation.

### 5.2. Regression results

Based on our experimental design, we performed anodal simulation of the left DLPFC and the right DLPFC compared to sham stimulation. Therefore, we conducted the regression models as mentioned in part 4. The regression analysis aimed to assess the tDCS stimulation influences of the left DLPFC and right DLPFC on attitudes about the participants’ choices regarding ambiguity loss and ambiguity gain. Tables 2, 3 show the results of the regression models. The results indicated the participants’ choices regarding ambiguity loss and ambiguity gain.

**Table 2:** The coefficients and significance of left DLPFC tDCS in the regression models.

Regressor	Base group: sham Coeff. (*p*)	
	Ambiguity loss	Ambiguity gain
L_anodal	−0.088* (0.351)	−0.280 (0.408)
Gender	0.572 (0.343)	1.195** (0.399)
Age	−0.031 (0.096)	0.062 (0.111)
Income	−0.037 (0.124)	−0.103 (0.144)
Adj R-squared	0.072	0.072
F-statistic	2.38	2.38
Observations	72	72

Standard error in brackets; ***p < 0.001, **p < 0.01, *p < 0.05.

**Table 3:** The coefficients and significance of right DLPFC tDCS in the regression models.

Regressor	Base group: sham Coeff. (*p*)	
	Ambiguity loss	Ambiguity gain
	Coeff.	Coeff.
R_anodal	−0.383 (0.359)	−0.192 (0.401)
Gender	0.527 (0.354)	1.191** (0.395)
Age	−0.044 (0.114)	0.009 (0.128)
Income	0.010 (0.134)	−0.009 (0.150)
Adj R-squared	−0.009	0.065
F-statistic	0.83	2.29
Observations	75	75

Standard error in brackets; ***p < 0.001, **p < 0.01, *p < 0.05.

As Table 2 shows, compared to the base group of sham stimulation, the left anodal stimulation had a significant effect on the participants’ attitudes toward ambiguity loss but had no significance on the participants’ attitudes toward ambiguity gain. Besides, there was a significant effect on gender toward ambiguity gain. However, the other control variables, such as age and income, had no significant effect on the participants’ attitudes toward both ambiguity loss and ambiguity gain. Furthermore, we find that the anodal stimulation parameters are negative, showing that there is a negative influence between the anodal stimulation and the participants’ attitudes toward ambiguity loss.

As Table 3 shows, compared to the base group of sham stimulation, the right anodal stimulation group had no significant effect on the participants’ attitudes toward ambiguity loss and had no significant effect on the participants’ attitudes toward ambiguity gain. In addition, the control variables, such as age and income, also had no significant effect on the participants’ attitudes toward both ambiguity loss and ambiguity gain. However, for the ambiguity gain regression, we find that there was a significant effect of gender.

### 5.3. tDCS results: Stimulation effect

Based on the regression analysis above, we found that the impact of stimulation of the left DLPFC on the participants’ attitudes toward ambiguity loss was robust *via* regression analysis. Then, we used a two-sample Wilcoxon rank-sum (Mann–Whitney) test to assess the relationship between the degree of the participants’ attitudes toward ambiguity loss/ambiguity gain and stimulation of left/right DLPFC activity.

**Figure 4:**
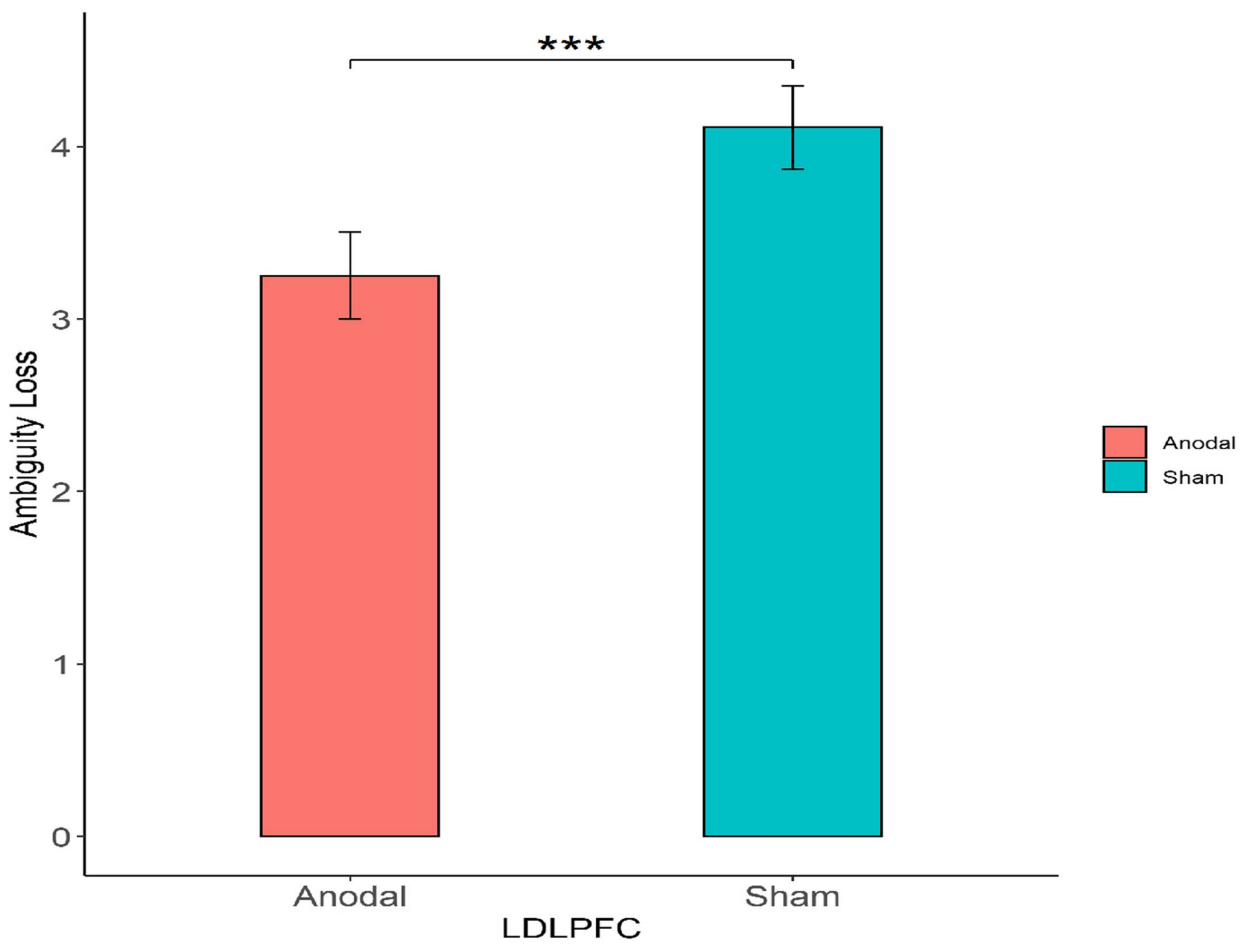
The degree of the participants’ attitudes toward ambiguity loss under different left DLPFC stimulation conditions. Error bars represent standard errors. Asterisks indicate statistically significant difference between the stimulation types.

**Figure 5:**
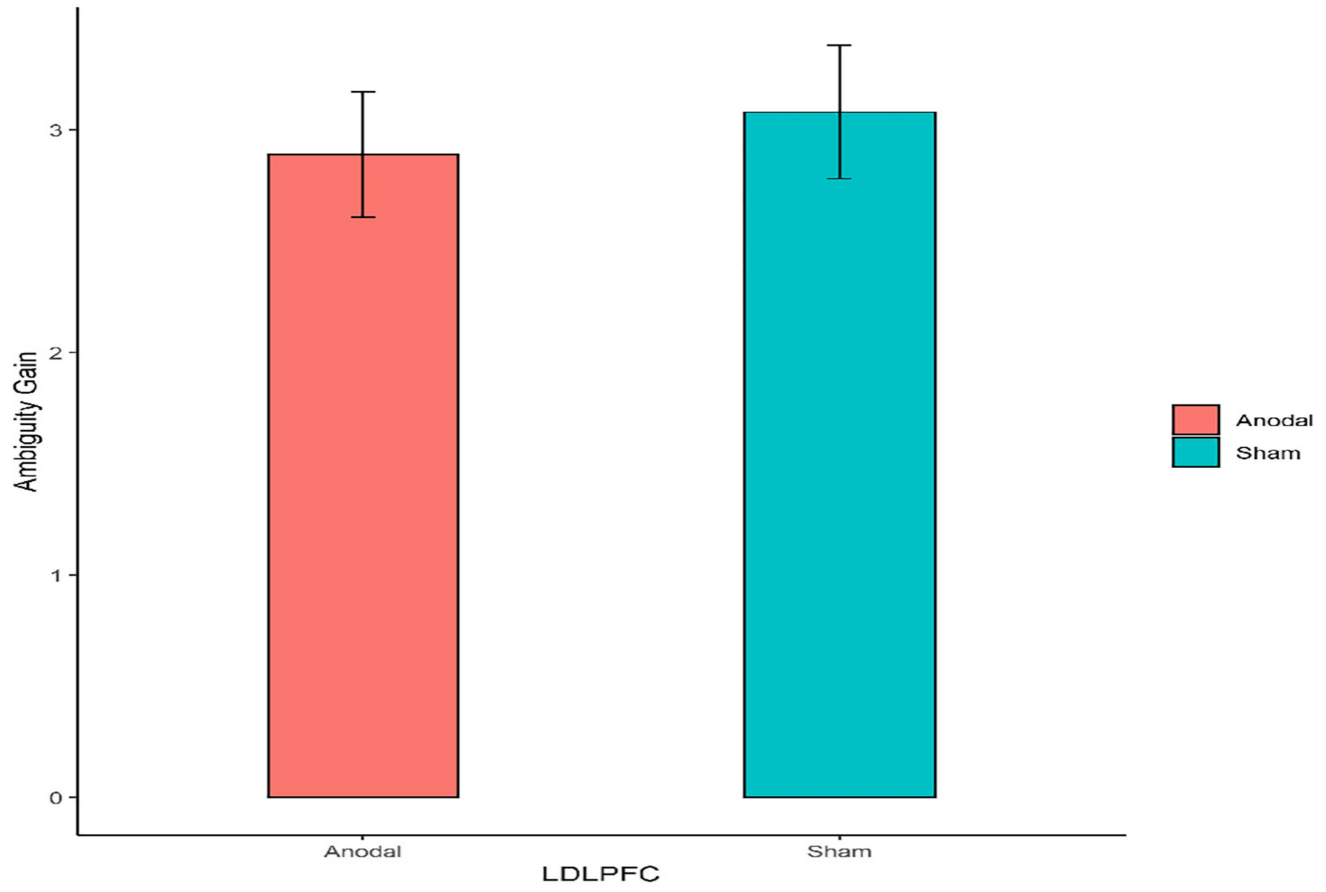
The degree of the participants’ attitudes toward ambiguity gain under different left DLPFC stimulation conditions. Error bars represent standard errors.

First, for the left DLPFC stimulation part, the Mann–Whitney test revealed that the degree of the participants’ attitudes toward ambiguity loss differed significantly between the anodal stimulation and sham stimulation (z = 2.501, *p* = 0.0124; Figure 4). The Mann–Whitney test revealed that there was no significant effect on the degree of the participants’ attitudes toward ambiguity risk between the anodal stimulation and sham stimulation (z = 0.332, *p* = 0.7401; Figure 5).

**Figure 6:**
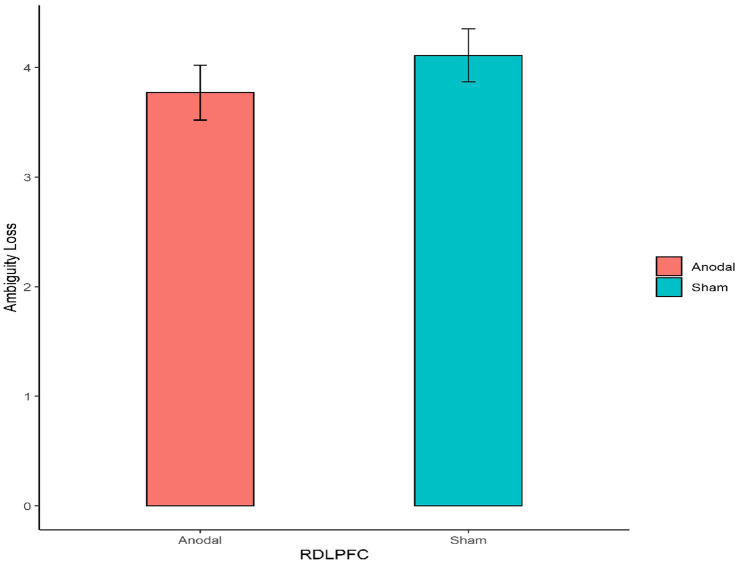
The degree of the participants’ attitudes toward ambiguity loss under different right DLPFC stimulation conditions. Error bars represent standard errors.

**Figure 7:**
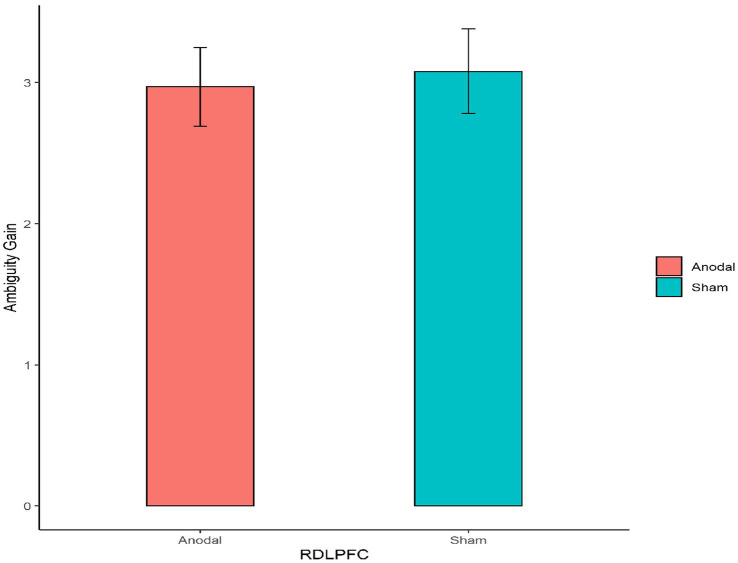
The degree of the participants’ attitudes toward ambiguity gain under different right DLPFC stimulation conditions. Error bars represent standard errors.

Second, for the right DLPFC stimulation part, the Mann–Whitney test revealed that there was no significant effect on the degree of the participants’ attitudes toward ambiguity loss between the anodal stimulation and sham stimulation (z = 0.910, *p* = 0.3626; Figure 6). In addition, the Mann–Whitney test revealed that there was also no significant effect on the degree of the participants’ attitudes toward ambiguity gain between the anodal stimulation and sham stimulation (z = 0.262, *p* = 0.7933; Figure 7).

Finally, we further found that the mean degree of the attitudes toward ambiguity loss among those who received anodal simulation (M = 3.25) was lower than that among those who received sham stimulation (M = 4.11) across the left DLPFC, which means that the subjects had a strong ambiguity loss aversion after receiving the anodal simulation of the left DLPFC.

## 6. Discussion

Based on our analysis, we found that there is a significant effect of left DLPFC stimulation on ambiguity to losses. However, the significant effect on left DLPFC stimulation about ambiguity to gain is not obvious, nor is that for right DLPFC stimulation about ambiguity loss decision and ambiguity gain decision. We also find that the degree of attitudes toward ambiguity loss among those who received anodal simulation is lower than that among those who received sham stimulation across the left DLPFC. From these results, there are three questions to ask: (1) Why is there a significant effect on ambiguity to loss but not to gain? (2) Why is the significant effect on left DLPFC obvious but that on right DLPFC not? and (3) Why is anodal stimulation lower than sham stimulation?

O'Doherty et al. (2001) and Smith et al. (2002), respectively, used brain imaging technology and positron emission tomography (PET) for their experiments, and it was found that people’s information processing of gain and loss was performed in different areas of the brain. Xu et al. (2009) studied the differences between the gains and losses of brain activity in the situation of cross-term selection by using brain imaging technology. They suggested that the human brain is more sensitive to future loss than to future gains, and this may be driven by negative emotions such as fear and disgust. Therefore, we conclude that in our experiment, compared to gains under ambiguity circumstances, people are more sensitive to losses under ambiguity circumstances because of human loss aversion. Loss aversion is the central element of prospect theory (Kahneman and Tversky (1979), Tversky and Kahneman, 1992) and the key to explaining deviations of expected utility (Rabin, 2000). There is abundant qualitative evidence from both the lab and the field experiment for loss aversion (Barberis, 2013).

The study performed by Krain et al. (2006) showed that the PFC, mainly the OFC and DLPFC, is engaged in decision-making under risky and ambiguous situations. Mohr et al. (2010) found that the right DLPFC is activated for risky decisions. In fact, the right DLPFC is involved in valuing the choice options during the decision-making process. Another fMRI study by Heekeren et al. (2006) demonstrated that the left DLPFC is activated during risky decision-making. Although it was hard to conclude these results for both sides of the DLPFC, Khaleghi et al. (2020) indicated that neural mechanisms in risk-taking behavior depend on the experimental task. They noted that their conclusion was limited to the technology of tDCS and BART tasks. Seibt et al. (2015) suggested that the left DLPFC, as a common target, is used in neuromodulation for therapy and cognitive enhancement, which play a role in rational approaches. Osaka et al. (2007) indicated that the left DLPFC played the role of executive function in human neuro mechanisms, which are involved in the processing of information. Therefore, for our experimental task of ambiguity loss, the left DLPFC played a role in executive function in processing the ambiguity information and loss information.

Khaleghi et al. (2020) indicated that the neural mechanisms in risk-taking behavior depend on the experimental task. Their research revealed that facilitation of left DLPFC activity, suppression of right DLPFC activity, suppression of left DLPFC activity, and facilitation of right DLPFC activity led people prefer to choose low-risk prospects. He et al. (2016) showed that modulating the activity of the left DLPFC could increase the Iowa Gambling Task (IGT) score while decreasing the recency parameter in the IGT. In other words, their study suggested that modulating the activity of the left DLPFC could improve people’s decision-making ability, and it could also make people more stable when making decisions. Therefore, when our participants received anodal stimulation on the left DLPFC, which means enhancing the activity of the left DLPFC, they became more stable in making decisions under ambiguity loss circumstances. Therefore, our results showed that the degree of attitudes toward ambiguity loss among those who received anodal simulation was lower than that among those who received sham stimulation across the left DLPFC. Overall, we can conclude that there is an obvious ambiguity loss aversion in human decision-making.

## 7. Limitations

From our results and existing results studied by other researchers, we found that the ambiguity decisions are impacted by different brain regions under different tasks. Even for the same task, the brain regions influenced ambiguity decisions may also be different. So, we think that it is not enough evidence to reveal the underlying mechanism of the results found in our study through tDCS technology. Future research can consider combining brain imaging with brain stimulation technology or other neuroscience technologies to study these problems. A recent research conducted by Xiong et al. (2021) showed that a preference for ambiguity can be measurably increased in individuals through right DLPFC anodal stimulation. The reason why this study is different from our research is that the subjects take part in different experimental tasks. Therefore, the future research can compare the differences of different experimental tasks through Meta-analysis of literature, which will help us to better understanding the mechanisms of brain areas about ambiguity decisions.

## 8. Conclusion

In our study, we performed an experiment to find a causal relationship between ambiguity and DLPFC activity. Our experimental tasks were designed by Cardenas and Carpenter (2013) and modified to adapt to our research purpose. The tasks were in the frame of ambiguity to gains and ambiguity to losses to distinguish the differences between gain and loss under ambiguity decisions. We used unilateral stimuli for the DLPFC by tDCS technology to target whether the left DLPFC or right DLPFC had aspected to the ambiguity decision-making. The results showed that there is a significant effect on left DLPFC stimulation about ambiguity to losses, which indicates that our participants have a stronger sensitivity to loss than to gain under ambiguity decision-making. However, the significant effect on left DLPFC stimulation about ambiguity to gain is not obvious, as well as right DLPFC stimulation about ambiguity loss decision and ambiguity gain decision. Furthermore, we find that the degree of attitudes toward ambiguity loss among those who received anodal simulation is lower than that among those who received sham stimulation across the left DLPFC, which suggests that individuals have a stronger ambiguity loss aversion when their activity of the left DLPFC is enhanced.

## Data availability statement

The original contributions presented in the study are included in the article/supplementary material, further inquiries can be directed to the corresponding author.

## Ethics statement

The studies involving human participants were reviewed and approved by the Zhejiang University of Finance and Economics Ethics Committee. The patients/participants provided their written informed consent to participate in this study.

## Author contributions

YH, XL, WZ, LW, and PY designed the experiment and wrote the manuscript. YH, XL, and PY organized the experiment. YH, WZ, and PY analyzed the data. YH, LW, and PY draw the figures. All authors contributed to the article and approved the submitted version.

## Funding

This research was supported by the National Social Science Foundation of China: Study on the Dynamics Mechanism and Path Optimization of Market Entry Reform of Rural Collective Management Construction Land in China (Grant number: 19BGL167), The key research base of philosophy and social sciences in Zhejiang Province: Research on the Boosting Mechanism of Sustainable Poverty Alleviation (Grant number: 2022JDKTYB17), The National Natural Science Foundation of China (Grant number: 72073117), the National Natural Science Foundation of China (Grant Number: 71903169), the Zhejiang Provincial Natural Science Foundation of China (Grant number: 19G030019), the Zhejiang Provincial Social Science Foundation of China (Grant number: 20JDZD021), and the Zhejiang Provincial Social Science Foundation of China (Grant number: 21GTFY03YB). The experiment was operated in the Center for Economic Behavior and Decision-Making (CEBD), Zhejiang University of Finance and Economics.

## Conflict of interest

The authors declare that the research was conducted in the absence of any commercial or financial relationships that could be construed as a potential conflict of interest.

## Publisher’s note

All claims expressed in this article are solely those of the authors and do not necessarily represent those of their affiliated organizations, or those of the publisher, the editors and the reviewers. Any product that may be evaluated in this article, or claim that may be made by its manufacturer, is not guaranteed or endorsed by the publisher.
